# Racial Differences in Immunological Landscape Modifiers Contributing to Disparity in Prostate Cancer

**DOI:** 10.3390/cancers11121857

**Published:** 2019-11-25

**Authors:** Jeronay King Thomas, Hina Mir, Neeraj Kapur, Shailesh Singh

**Affiliations:** 1 Department of Microbiology, Biochemistry and Immunology, Morehouse School of Medicine, Atlanta, GA 30310, USA; Jkthomas@msm.edu (J.K.T.); hmir@msm.edu (H.M.); nkapur@msm.edu (N.K.); 2 Cancer Health Equity Institute, Morehouse School of Medicine, Atlanta, GA 30310, USA

**Keywords:** racial disparity, immunity, African American, Caucasian

## Abstract

Prostate cancer affects African Americans disproportionately by exhibiting greater incidence, rapid disease progression, and higher mortality when compared to their Caucasian counterparts. Additionally, standard treatment interventions do not achieve similar outcome in African Americans compared to Caucasian Americans, indicating differences in host factors contributing to racial disparity. African Americans have allelic variants and hyper-expression of genes that often lead to an immunosuppressive tumor microenvironment, possibly contributing to more aggressive tumors and poorer disease and therapeutic outcomes than Caucasians. In this review, we have discussed race-specific differences in external factors impacting internal milieu, which modify immunological topography as well as contribute to disparity in prostate cancer.

## 1. Introduction

Cancer-associated racial disparities have been recognized by the scientific community since the early 1970s. African Americans (AAs) are more likely to develop cancer and show worse prognosis compared to Caucasian Americans (CAs) [1,2]. Differences in the incidence and outcome of cancer among different ethnicities add a layer to an already complex disease. The fact that standard treatment options offered in clinics fail to provide similar outcomes in different ethnic groups suggests that differences in the biology of cancer contribute to racial disparities in therapeutic outcome. Moreover, several inter-related factors, such as socio-economic status (SES), lifestyle, and various cultural aspects influence biology and physiology. These factors together affect the probability and course of disease progression, while differences in these aspects maintain race-specific gaps in incidence, progression and therapeutic outcomes of cancer. Intriguingly, even after normalizing for the socio-economic factors and healthcare inequalities, racial disparities in disease and therapeutic outcome exist.

Studies have shown that Gleason score and levels of prostate specific antigen (PSA), a biomarker for prostate cancer [2,3,4], may be higher in AA prostate cancer (PCa) patients in comparison to their CA counterparts. Metastatic progression [5] and recurrence [6] is higher in AA men in comparison to other Americans. In addition, localized advanced stage PCa is potentially more aggressive in AA men in comparison to CA [7]. They are two times more likely to die of PCa than CA men [1,8]. In 2019, 202,260 new cases and 73,030 cancer-related deaths were expected to occur among AAs, with PCa being the most common cancer among AA men [1]. This disparity in mortality increases to greater than fourfold in younger men [8]. Furthermore, AA men are more likely to be diagnosed at a younger age than CA man [8,9]. Post-treatment progression-free survival is shorter in AA in comparison to CA [10]. Even after prostatectomy, AA patients have a lower three-year disease-free survival rate than CA men [2]. Worldwide incidence of PCa has been suggested to be highest in African and Jamaican men [11,12]; therefore, this observed disparity is likely attributed to predisposed genetic susceptibility.

This emphasizes the significance of addressing the differences in host factors in improving our understanding of this multifactorial issue. Studies related to HIV, birth outcomes and graft/transplant have suggested immunological differences among AAs and CAs [13,14,15,16,17,18]. Immunology and oncology have a longstanding relationship. Therefore, in this review, we discuss the race specific differences in host factors, which define the immunological landscape in PCa, and their impact on PCa racial disparity.

### 1.1. Immune System and Cancer

Genetic diversity in the world’s population is largely attributable to geographic differentials in allele frequencies [19,20]. Populations of African descent have more nucleotide diversity and rare alleles when compared to populations of European and Asian decent [20]. Specifically, immune adaptations suited to ancestral environments influence cancer risk between AAs and CAs [21,22], implying that the erosion in adaptive and innate immune surveillance could be a key player in PCa disparity.

### 1.2. Innate and Adaptive Immune Surveillance

Hosts’ internal defense systems consist of innate and adaptive immune responses. The innate response is immediate, less specific, and is followed by the antigen-specific adaptive response [23]. Complement systems function to remove infectious particles out of the body and bridges the innate and adaptive arms of the immune system [24,25,26]. This intricate system orchestrates the anti-tumorigenic actions by direct elimination of tumor cells based on the expression of tumor-specific antigens (TSAs) and by suppression of viral infections protecting the host from virus-induced tumors [27]. It prevents formation of an inflammatory microenvironment conducive for tumorigenesis (acute inflammation is a characteristic of cancer) [28,29,30,31]. Compelling evidence using carcinogen-induced and spontaneous cancer in immune-deficient mice suggests synergistic involvement of both innate and adaptive immune systems in preventing cancer development [32,33,34,35]. Simply put, developing (non-syngeneic) tumors in immune competent hosts is very difficult, and immunosurveillance is the most important process by which a healthy host gets rid of transformed cells.

**Figure 1:**
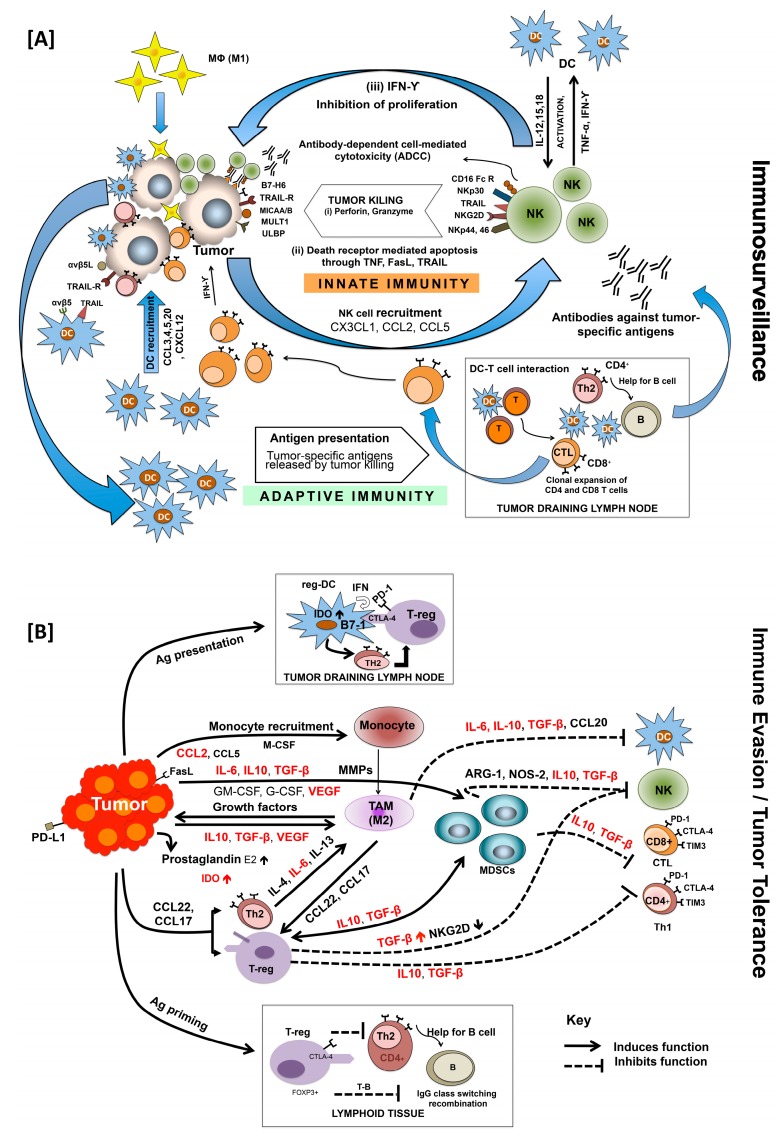
Immunosurveillance, evasion, and tolerance in cancer. Innate and adaptive immune systems work in consortium to eliminate cancer cells before their clinical appearance (**A**). Innate immune cells (NK) interact with neoplastic cells through their surface receptors (NKG2D, NKp30, NKp44, NKp46, CD16) and kill them by (i) release of cytotoxic granules (perforin, granzyme) in the vicinity; (ii) death (TNF, FasL, TRAIL) receptor-mediated apoptosis; and (iii) secretion of IFN-ϒ which inhibits proliferation of tumor cells by activating M1 macrophages (Mϕ) and DCs as well as Th1 cells of adaptive immune system. Dendritic cells (DCs), recruited at the tumor site, present tumor-specific antigens released by tumor killing. Antigen presenting DCs interact with naive T-cells in tumor draining lymph nodes facilitating clonal expansion of CD4+ and CD8+ T-cells which then differentiate into antigen-specific effector T-cells: T-helper cells (Th1, Th2, Th17) and cytotoxic T-cell lymphocytes (CTL), respectively. DCs also control the humoral part of adaptive immunity either by directly interacting with B cells or through CD4^+^ helper T-cell by differentiating B cells into antibody secreting cells. In addition to immune surveillance failure, cancer progress by evading immune attack (**B**). Immune pressure selects poorly immunogenic tumor cells, not recognized by effector cells of innate and adaptive immunity. These immune-evasive cells modulate TME further to make it more immunosuppressive by activating accessory cells: regulatory T-cells (T-regs), tumor-associated macrophages (TAMs), regulatory dendritic cells (reg-DCs), and myeloid-derived suppressor cells (MDSCs). The combined activity of these immune suppressor cells regulates tumor growth, survival, migration, and invasion by changing the hormone, growth factor, and cytokine profile of TME. Levels of cytokines involved in immune suppression and evasion and which are higher in AA are highlighted in red.

Natural killer (NK) cells are among the first innate immune cells that respond to inflammation and cancer. Activated NK cells eliminate target cancer cells by either secreting perforin or granzyme or by apoptosis mediated through interaction of TNF, FasL, and TRAIL with corresponding death receptors (Figure 1A) [36]. Activation of NK cell depends on the balance of their activating (NKG2D) and inhibitory receptor (KIR). Cytokines, such as IL-2, IL-12, IL-15, IFN-α, and IFN-β, are required for NK cell activation [36,37]. Tumor cell killing by NK cells offers a TSA-enriched environment for dendritic cells (DCs), another subset of innate immunity that plays an important role in tumor cell clearance, either directly or through activation of adaptive immune responses. Several complementary mechanisms help DCs to capture NK cell-induced TSAs, which are then processed and bound to MHC-I/II molecules during their journey to draining lymph nodes (DLNs) [38]. Besides, TSAs can also directly reach lymph node-resident DCs through lymph [39]. These antigen-loaded lymph node-resident DCs are the first to present antigenic peptides to naïve CD4+ T-cells leading to T-cell priming and IL-2 production, facilitating their proliferation and expansion. Subsequently, activated DCs from peripheral tissues migrate to lymph nodes and interact with these activated CD4+ T-cells, facilitating their conversion to effector T-cells (Figure 1A). It is important to mention here that immature, non-activated DCs that present self-antigens to T-cells bring about immune tolerance by T-cell deletion or by suppressor T-cell differentiation [40]. Upon interaction with mature DCs, naïve CD4+ T-cells differentiate into effector T-cells that include T-helper (Th1, Th2, Th17 or T-follicular helper (Tfh)) cells. These cells help in differentiation of B-cells into antibody-secreting cells as well as in generation of regulatory T (Treg) cells. Naïve CD8+ T-cells, on the other hand, differentiate into effector cytotoxic T-lymphocytes (CTLs). Tissue-localized DCs can also be polarized into distinct effector phenotypes by interaction with other cells of innate immunity via the release of IFNs, TNFs, and other cytokines [41,42,43,44,45,46,47]. Much like DCs, macrophages are phagocytic cells that reside in many tissues, recognize TSAs, and produce high levels of cytokines including chemokines that function as the alert signals for the immune system [48]. Activation of innate immunity promotes various inflammatory reactions and triggers the release of inflammatory cytokines as well as other inflammatory mediators [48,49]. In context to cancer, there are reports suggesting anti- as well as pro-oncogenic roles of innate immunity. Contrary to the physiological role, a plethora of evidence suggests the role of the immune system in the establishment of tumor cells and their subsequent progression. This, however, is due to the manipulation of the immune system by transformed cells, their escape from immune surveillance, and which eventually makes the disease clinically significant [50]. All this raises questions regarding the effectiveness of immunity against spontaneously arising tumors, spiking interest in immunotherapy for cancer [51].

### 1.3. Immunological Landscape and Cancer Progression

Cancer develops either by remaining less immunogenic and unnoticed or by bending the immune system for its benefit. This dynamic “cancer immune-editing” process is characterized by changes in the immunogenicity of tumor cells along with changes in the immunological landscape leading to escape from immune surveillance. Low antigen expression, recruitment of suppressive immune cells, and production of immune modulating factors, ultimately resulting in alteration of the immune microenvironment are some of the mechanisms by which tumors escape immune attack [24,25,26,52]. Cancer immunoediting begins with clonal selection of less immunogenic cancer cells while the innate and adaptive immune system eliminates the more immunogenic cells. This results in the immune selection of poorly immunogenic tumor cell variants and eventually supporting cancer progression by the following “immune sculpting” phase. During immuno-sculpting, selected cancer cells that are tolerant of immune attack, after exposure to the effector immune cells, steer the immune response by production of selective cytokines for recruitment of suppressive immune cells and facilitate proliferation after escaping elimination [53].

Myeloid-derived suppressor cells (MDSCs), regulatory T-cells (Treg/CD4+CD25+FOXP3+), and tumor-associated macrophages (TAMs) (Figure 1B) are tumor-modified immune cells that hinder the clearance of cancer cells locally and even in distant organs [53,54]. Myeloid-derived suppressor cells are composed of a heterogeneous population of immature myeloid cells that abrogate innate and adaptive immune responses [55,56,57,58]. Immature myeloid cells give rise to macrophages, DCs, and granulocytes, all of which are essential for normal function of the immune system; however, these cells can be converted into potent immune suppressors in a tumor microenvironment (TME) [59]. Expansion of a pool of immature myeloid cells is capable of inhibiting an antigen-specific CD8+ T-cell response in cancer patients and tumor-bearing mice [58]. Myeloid-derived suppressor cells suppress T-cell proliferation and cytotoxicity, induce the expansion of Tregs, and block NK cell activation by expressing suppressive factors such as arginase-1, ROS, and iNOS [60]. Treg cells are normally responsible for preventing excessive immune reactivity and maintaining the immune balance [60] but also support tumor progression [61]. They inhibit the activity of a variety of immune cells through contact-dependent mechanisms or by secreting immunosuppressive cytokines [60]. Tregs are capable of inhibiting the anti-cancer functions of T-cells [62], NK cells [62], and B-cells [63]. Additionally, TAMs are a subset of macrophages which play a significant role in tumor immune evasion, angiogenesis, and metastasis. Evidence suggests that macrophages polarize into immunosuppressive M2 macrophages upon exposure to M2 differentiation factors produced by the tumor immune microenvironment (TIME) [64]. These macrophage subset can contribute to cancer progression by producing soluble factors like TGF-β and IL-10 that promote tumor growth and/or help tumor cells evade from host immune surveillance [64,65,66,67,68,69]. Lastly, cancer-associated fibroblasts (CAFs), also known as activated fibroblasts, myofibroblasts, or tumor-associated fibroblasts, have been investigated for their pro-tumorigenic capabilities [70]. These cells have recently been reported to modulate the immune system through secretion of TGF-β and other immune suppressive cytokines, creating an immunosuppressive environment [70]. All these changes in the TIME are brought about by combined actions of hormones and cytokines secreted by different cells in and around the tumor.

### 1.4. Racial Differences in Hormonal Status Impacting Immune System and Prostate Cancer

Steroid hormones significantly affect immunity by regulating the activity of lymphocytes (T- and B-cells), monocytes, and NK cells [71]. In fact, there is a direct correlation between a healthy immune response and hormonal balance of the body. Interplay of sex hormones and immunity is well studied and is rendered responsible for sex-based differences in immune responses [72,73]. Thus, immune response against cancer is also highly influenced by hormonal milieu and vice versa. 

Expression of androgen receptors (ARs) by various immune cell lineages [74,75,76] and the fact that androgen plays a major role in the regulation of both innate and adaptive immune response [77] implies that differences in androgen signaling could also lead to PCa disparity. It regulates the proliferation, maturation, function, and chemokine-mediated recruitment of neutrophils. Deficiency in ARs leads to significant neutropenia, while insufficient testosterone causes a mild reduction in neutrophils. Testosterone dampens the activation of macrophages and DCs as well as suppresses the production of pro-inflammatory cytokines (TNF-α, IL-1β, IL-6) by these cells [77,78,79]. On the other hand, it promotes activation of regulatory MDSCs and Tregs [80]. Castration-based studies also revealed a significant increase in MHC-II expression and co-stimulatory molecules by cDCs (cytotoxic DC) [81]. Further, androgen deprivation causes thymic enlargement in males, facilitating an increased peripheral T-cell population [82,83,84,85,86,87].

The level of cytokines that governs the Th1:Th2 ratio determines the risk of several types of cancer. A higher Th1:Th2 ratio prevents tumor development, whereas a Th2 polarized immune system promotes tumor development. Androgen (testosterone) inhibits Th1 differentiation as well as production of pro-inflammatory cytokines (IFN-γ, TNF-α/β) and decreases IL-12-mediated induction of Th1 phenotype [88]. Th effect of androgen on Th1 differentiation is governed by upregulation of Ptpn1 (protein tyrosine phosphatase, non-receptor type 1) that is involved in IL-12-induced phosphorylation of STAT4 via inactivation of Jak2 and Tyk2 kinases [88,89]. PCa patients undergoing androgen ablation therapy have decreased expression of Ptpn1 in their T-cells, further showing a direct correlation between androgen and Th1 response. Testosterone is also shown to promote Th2 differentiation and IL-10 production by these cells. In turn, IL-10 has an anti-inflammatory effect and facilitates the expansion of Tregs. Altogether, these changes suppress anti-tumor immune response [90,91,92,93]. In addition to favoring PCa by impacting Th1/Th2 imbalance, this male sex hormone also regulates humoral immune response by affecting B-cell development in a negative manner. Testosterone promotes TGF-β secretion from bone marrow stromal cells which reduces IL-7-mediated proliferation and differentiation of B-cells [76,82,94,95]. Therefore, a higher androgen level impacts PCa outcome negatively. This is also evident from the studies that demonstrate that treating lymphocytes from female mice with testosterone weakened their proliferative response to antigens and caused poorer antigen presentation capability in comparison to normal controls [80,96,97]. While females having significantly lower levels of testosterone are more prone to autoimmune diseases than men, hypogonadism in men leads to increased incidence of autoimmunity implying the immunosuppressive role of androgen [72,73,98,99]. Thus, testosterone plays a major role in cancer incidence and prognosis by manipulating the immune system making males more susceptible to cancer and secondary malignancies compared to females [76,82,94,95,100]. Elevated testosterone levels have not only been shown to increase the risk of sex-specific cancers, but the male–female incidence rate ratio is also high in cancers not specific to sex. Although there are few conflicting reports, PCa risk and development have been linked to elevated testosterone levels [98,99,100,101]. Of particular interest is the fact that AA men have higher circulating testosterone concentrations, along with a greater DHT/testosterone ratio when compared with CA men, which may explain the underlying differences in PCa incidence among these two races [102,103,104,105,106,107]. Furthermore, AAs have increased sex hormone-binding globulin, which increases production of cAMP, a co-activator of the testosterone receptor [108,109,110]. However, contradicting studies about significant difference in serum testosterone levels between AA and CA men exist [111,112]. This also signifies the importance of studying androgen independency observed in castration-resistant prostate cancer (CRPC). This will allow fishing out biomolecules (like cytokines, chemokines and growth factors) that serve as alternate mediators/activators of AR signaling and which are different between the two racial groups.

In addition to testosterone, glucocorticoid also modulates immune function. It can act as an immune stimulant however, when it is consistently high, as found with low SES [113], it becomes immune suppressive. Those AAs belonging to lower SES will have increased cortisol and hence will be more prone to tumor-induced immune suppression. Glucocorticoid levels regulate transcription of various genes, such as histone acetylation in the promoter region of *perforin* and *granzyme B*, thereby affecting NK cell cytotoxicity [114] as well as CTLs. Thus, changes in this hormone will have a global effect on host immunity [115]. It is also known that high glucocorticoid levels lead to the resistance of immune cells to immune stimulants that further deteriorate defense against cancer. Considering the structural similarity and, hence, the shared target genes between glucocorticoid and androgen receptors, it would not be surprising if glucocorticoid underlies castration resistance in PCa [116] and its associated disparities.

Vitamin D and its derivatives are another closely related class of hormones, which could significantly impact cancer-immune regulation. Also, the fact that almost all immune cells, including monocytes, macrophages, DCs, and activated lymphocytes, express VDR (Vitamin D receptors) clearly suggests an immuno-modulatory role of Vitamin D. Vitamin D exerts its effects on immunity, in addition to regulating calcium and bone homeostasis, by binding of its biologically active metabolite 1,25-dihydroxyvitamin D3 (1,25(OH)-2D) to specific intracellular VDR. 1,25(OH)-2D inhibits the production of inflammatory cytokines, such as IL-1, IL-6, IL-8, IL-12, and TNF-α, by monocytes [117]. It prevents differentiation and maturation of DCs, restricting them to their immature phenotype as evidenced by the reduced expression of MHC-II molecules, co-stimulatory molecules, and IL-12 [118]. However, at local chronic inflammation sites, elevated 1,25(OH)-2D levels augment IL-1 production and MHC-II expression in an autocrine or paracrine fashion thereby enhancing antigen presentation by tissue monocytes/macrophages. Interestingly, high levels of 1,25(OH)-2D also adversely affect adaptive immunity. It inhibits B-cell proliferation and differentiation, suppresses T-cell proliferation, decreases the Th1:Th2 ratio leading to reduced inflammation, shifts away from inflammatory Th17 phenotype, and enhances the development and function of Treg cells [119,120,121,122,123]. These pleotropic effects of Vitamin D culminate in increased production of anti-inflammatory cytokines (IL-10) and decreased production of inflammatory cytokines (IL-21, IL-17). Vitamin D supplementation suppresses *IL-6, IL-8* and *TNF-α* in prostate epithelial cells while expression of *TNF-α* and *PTGS2* (COX-2) is greatly reduced in stromal cells [124]. 1,25(OH)-2D inhibits NF-κB by inducing IκBα; this, in turn, prevents subsequent expression of *IFN-β* and *CXCL10* [125]. Based on its preferential suppressive effect on CXC chemokines, Vitamin D is suggested to reduce recruitment and activation of T-cells [126]. Thus, deficiency of Vitamin D in cancer patients may affect macrophage infiltration by regulating the expression of chemokines in adipocytes.

Despite the well-established immunomodulatory function of Vitamin D on a variety of immune cells and the experimental evidence suggesting an association of lower Vitamin D levels with cancer prognosis and anti-proliferative action on cancer cells, direct correlation with anti-cancer immunity are scarce. Occurrence and mortality rates of bladder, breast, colon, endometrial, lung, ovarian, pancreatic, prostate, rectal, testicular, vaginal cancer, Hodgkin lymphoma, and melanoma negatively correlate with serum Vitamin D [127]. Particularly, in the case of PCa, reduced serum Vitamin D is associated with advanced stage, higher tumor grade, and mortality [128,129,130,131]. Levels of PTGS2 that are significantly higher in PCa are suppressed with 1,25(OH)-2D treatment [132,133,134]. 1,25(OH)-2D also inhibits NF-kB signaling by preventing its interaction with DNA response elements responsible for IL-8 production, suppressing angiogenesis in PCa [135]. Since, in healthy prostate, Vitamin D inhibits the production of pro-inflammatory cytokines responsible for PCa initiation and subsequent progression, chronic Vitamin D deficiency in AAs may create a pro-inflammatory TME that may be responsible for aggressive PCa in these patients compared to their CA counterparts [136,137]. Nonetheless, Vitamin D-mediated molecular pathways and associated inflammation in PCa still need to be explored.

### 1.5. Racial Differences in Cytokine Profiles in Prostate Cancer 

Cytokines are hormone-like messengers which act to regulate the development and expression of a broad array of immune responses described above. These molecules serve as means of communication in coordinating the adaptive and innate immune response. These are a heterogeneous group of soluble small proteins (5–20 kDa) including interleukins (ILs), interferons (IFNs), tumor necrosis factors (TNFs), colony-stimulating factors, growth factors, and chemokines. Many of the key drivers of neoplastic progression, such as neutrophils, MDSCs, TAMs, and Tregs cells, work by secretion of pro-inflammatory cytokines, including IL-1, IL-6, TNF, and TGF-β (Figure 1B), providing a basis for a link between inflammation and cancer [138,139,140,141,142,143]. Several cytokine polymorphisms have been associated with cancer incidence [144]. Alleles associated with increased cytokine production are more frequently found in AA [145,146,147,148,149,150].

Pro-inflammatory cytokine, IL-6, is involved in the regulation of various cellular functions, i.e., proliferation, apoptosis, angiogenesis, differentiation, and regulation of immune response. It is thought to be associated with faster tumor progression, decreased effectiveness of therapy, increased relapse, and decreased survival. Indeed, the poor outcome of many cancer patients is closely associated with elevated serum levels of IL-6. Enhanced IL-6 signaling has been found to be responsible for cancer development and tumor progression in many human cancers including lung, liver, breast, ovarian, pancreatic, prostate, glioma, lymphoma, melanoma, renal, and colorectal cancers [151,152]. It has also been reported to play a key role in chemoresistance in most cancers by maintaining residual tumor cells causing tumor relapse. Its expression can also be linked to the stage, size, and metastasis of tumors affecting the overall survival of the patients. Level of IL-6 also correlates with SES and it significantly differs among healthy AAs and CAs [153]. Serum IL-6 level-based cancer prognosis in the Multi-Ethnic Cohort Study revealed association with significantly poor survival in AAs (Hazard ratio: 2.71) compared to CAs (Hazard ratio: 1.71) [154,155]. Gene expression profiling showed significant differences in levels of pro-inflammatory cytokines (IL-1β, IL-6, and IL-8) in AA and CA PCa patients, which potentially accounts for the observed disparity in PCa. Besides, stromal compartment also showed differential expression of many immune-related genes, mainly involved in cytokine-mediated pathways [156]. In fact, Giangreco et al. found ~18 fold higher IL-6 expression in PCa-associated stroma compared to benign epithelium [124]. This inflammatory microenvironment of stroma regulates the differentiation and proliferation of PCa epithelial cells and also mediates immune response. Probably, a heightened pro-inflammatory stromal microenvironment is responsible for aggressive PCa in AAs compared to CAs. Moreover, chronic inflammation may set the stage for epigenetic changes and genomic instability which may further promote aggressive PCa in AAs. The frequencies of alleles responsible for upregulating pro-inflammatory cytokines are significantly higher in AAs than in CAs. The human *IL-6* gene on chromosome 7p21-24 has a common G/C polymorphism of the *IL-6* promoter region on position −174 upstream of the transcription start site that impacts serum cytokine levels [157,158]. Data demonstrate a strong association of the −174 G/C polymorphism with the aggressiveness and recurrence of PCa [159]. Higher IL-6 levels and, hence, IL-6 transcription activity was found with G allele homozygosity compared to C allele homozygosity [160]. It is reported that the *IL6*-174 G/G genotype was much more common among AAs than CAs [147,148,149]. One report suggests that the −174 C/C genotype in AAs could be a strong predictor of aggressive metastatic disease, whereas the G/G genotype in this racial group could suggest an increased risk of cancer [161]. Further, IL-6 has been identified as a major regulator of the balance between regulatory Treg and T helper cells [162]. It also regulates initiation and maturation of Th2 cells along with IL-4 [163]. Elevated IL-6 has been shown to contribute to TAM infiltration [164] and MDSC induction [165] (Figure 1B). It maintains the pro-tumorigenic milieu of immune cells in the TME by supporting angiogenesis as well as evasion of immune surveillance. Additionally, IL-6 induces the transcription of C-reactive protein (CRP) [166], an inflammatory effector that has been reported to have higher circulating levels observed in AAs [167,168]. Over a period of time, these heightened levels have been linked to poor clinical outcomes and increased incidence in various malignancies. In contrast, Heikkilä K et al. [169] conducted a systematic review on CRP and found that CRP did not play a causal role in cancer. Evidence highlighting the anti-tumor role of IL-6 associated with maneuvering of T-cell immunity has come up [59,139,142,144,170,171,172,173,174,175,176]. However, it is the pro-tumorigenic role of IL-6 that links chronic inflammation to tumorigenesis and subsequent metastatic progression [147,148,149,150] that becomes the major factor governing PCa disparity.

Promoter polymorphism of IL-10 may influence tumor development by altering its levels in serum or the TIME. Elevated IL-10 in cancer is associated with TAM infiltration (Figure 1B), downregulating pro-inflammatory cytokines, MHC class II molecules, and co-stimulatory proteins [177,178,179]. The prominence of cancer risk related to *IL-10* alleles and its protein expression is not fully understood and varies among different types of cancers [180]. For example, the *IL-10*−592 polymorphism is associated with a protective effect against non-cardia gastric cancer [181]. There was no effect of the *IL-10*−1082 G/A polymorphism on cervical cancer risk but there was a clear association with the *IL-10*−592 C/A polymorphism [182]. In BrCa, the *IL-10*−592 A/A is associated with reduced susceptibility [183], whereas in PCa, *IL-10*−819 C and *IL-10*−592 C polymorphisms may be associated with aggressiveness [184]. Genotypes (*IL-10*−819 T/T, *IL-10*−592A/A, and *IL-10*−1082 A/A) that are associated with lower IL-10 production are three-fold higher in AAs [149,150]. To the contrary, AAs may express higher levels of the allele (−3575T) associated with increased production of IL-10 [147,148,185] when compared to CAs.

Apart from interleukins, interferons are another important set of immune regulatory cytokines that affect immune responses. Though conventionally known for its anti-tumor properties, interferon-gamma (IFN-γ) has pro-tumorigenic roles as well [186]. The contrasting roles of IFN-γ depend on cellular and microenvironmental factors. It plays an important role in tumor surveillance by upregulating MHC-I and thereby increasing immunogenicity of cancer cells. It activates macrophages to secrete chemokines which recruit cytotoxic T-lymphocytes (CTLs) to the site of inflammation culminating into tumor elimination. However, the cancer-promoting role of IFN-γ is attributed to its suppressive effects on CTL and NK cell activity. It also attenuates myeloid cell and neutrophil infiltration into TIME [186]. Studies have found that AAs do not differentially express *IFN-γ* when compared to other populations; however, there are significant differences in allelic and genotypic frequencies [148]. When compared to CAs AAs are more likely to express the *IFN-γ* AA genotype [187,188]. This genotype is associated with decreased IFN-γ production [189,190,191]. However, several studies have reported higher mRNA expression of *IFN-γ* in AAs [147,192,193]. Hence, the precise role of IFNs in PCa disparity is debatable at present and needs further investigation.

Of note is the fact that cytokines associated with both Th1 and Th2 are found to be very high in African adults as opposed to their European counterparts [194]. Also, variants in the Th1-related cytokine genes (*IL-12β* and *IFN-γ*), which reduce the severity of malaria, are likely among AAs [195,196]. Single Nucleotide polymorphisms associated with Th1-related cytokines and their receptors *IL-15*, *IL-15RA*, and *IFNGR2* have been found associated with BrCa risk in AA women [197]. Interestingly, Kimball et al. [198] found that AAs have a greater Th1 response but less IL-10 than healthy CAs in a hepatitis-related study.

Another cytokine that acts as a double-edged sword, having both a tumor suppression as well as progression role, is TGF-β. It regulates tumor growth by arresting cells in G1 phase during the early stages of tumorigenesis [199] and inhibits the clonal expansion and cytolytic activity of NK cells and CTLs (Figure 1B) thereby promoting tumor progression [200]. Signaling triggered by TGF-β also plays a significant role in immunoediting by promoting a Th2 phenotype of T-cells. It also channelizes MDSCs to secrete pro-angiogenic chemokines (Figure 1B) [201,202,203,204,205,206]. Evidence also suggests that TGF-β may promote tumorigenesis by inducing epithelial-to-mesenchymal transition, interfering with cell adhesion, and increasing cell invasiveness. Increased expression of the isoform *TGF-β3* has also been speculated to contribute to the migration and invasion of PCa cells. More specifically, evidence confers that TGF-β is an important promoter of malignant cell growth [207,208]. It upregulates *Foxp3* which is essential for the development and function of Tregs (Figure 1B) [209,210] and it is also capable of hampering the function of DCs (Figure 1B) [211]. There is a great deal of evidence suggesting the overexpression of TGF-β among AAs [212,213,214,215,216,217]. Profiling of TGF-β1 in AAs revealed overexpression of circulating proteins as well as mRNA levels compared to their CA counterparts. This implies that AAs have a more immunosuppressive environment compared to CA, which may contribute to disparity in PCa.

Vascular endothelial growth factor VEGF, a proficient angiogenic cytokine, is also known for its immunosuppressive function. Hypoxia-induced HIF-1α (hypoxia-induced factor-1α) in the TIME is a principle regulator of VEGF expression by monocytes. Hypoxic conditions also upregulate the expression of pro-angiogenic as well as pro-inflammatory factors (TNF, IL-1, IL-6, and IL-8) via HIF-1α and NFκB signaling [218]. The release of IL-6, IL-8, and CXCL1 from endothelial cells is enhanced by VEGF through an autocrine effect thus creating a pro-inflammatory environment responsible for tumor progression [219]. Interestingly, the expression of VEGF can itself be induced by inflammatory cytokines: TNF-α, IL-1β, IL-6, and IL-8, suggesting a positive feedback loop [218]. It induces *COX2* (cyclooxygenase2) expression in endothelial cells via p38 and JNK pathways [220]. Further, it also acts as a chemo-attractant for macrophages in the TME, and these tumor-guided macrophages are themselves a good source of VEGF, MMPs, and M-CSF/CSF1 (Figure 1B). Thus, VEGF contributes significantly in the development of immune tolerance. African Americans have significantly high VEGF [221,222], which may be the reason why AA PCa patients develop immune tolerance sooner than CAs and suffer more aggressive PCa.

### 1.6. Immune-Based Strategies Available to Treat Prostate Cancer and Their Impact on Reducing Disparity

With the current knowledge of disarrayed host immune systems contributing significantly to PCa progression, outcome, and associated disparity, novel therapies are being designed to overcome immune tolerance, restore Th1 response, and activate CTL to treat PCa. Although long-term studies are imperative, the initial results of these immune therapies sound promising. A CAR–T-cell therapy targeting prostate-specific membrane antigen-II (PSMA-II) showed promising results in a PCa mice model [223]. A phase I CAR-T-cell clinical trial targeting PSMA after non-myeloablative conditioning and IL-2 administration showed reduction in PSA levels [224]. Another phase I CAR–T-cell therapy directed against PSMA showed tolerability and systemic persistence of about two weeks [225]. However, the most successful and recently approved sipuleucel-T, an autologous cellular immunotherapy for CRPC, reduced the risk of death among patients with metastatic CRPC and improved the median overall survival (OS) by 4.1 months versus a placebo in the pivotal phase of the three-trial Immunotherapy for Prostate Adenocarcinoma Treatment study (IMPACT; NCT00065442) [226]. Remarkably, AA men showed an unexpectedly higher survival advantage in the PROCEED trial for receiving immunotherapy for metastatic CRPC [227]. As compared to a placebo, sipuleucel-T led to an overall survival (OS) of 20 months based on the randomized trial [228] with OS 9.3 months longer in AAs than CAs. Such better response by AAs further substantiates the significance of considering immunological differences in patients of African Ancestry before determining the treatment course.

## 2. Conclusions

**Figure 2:**
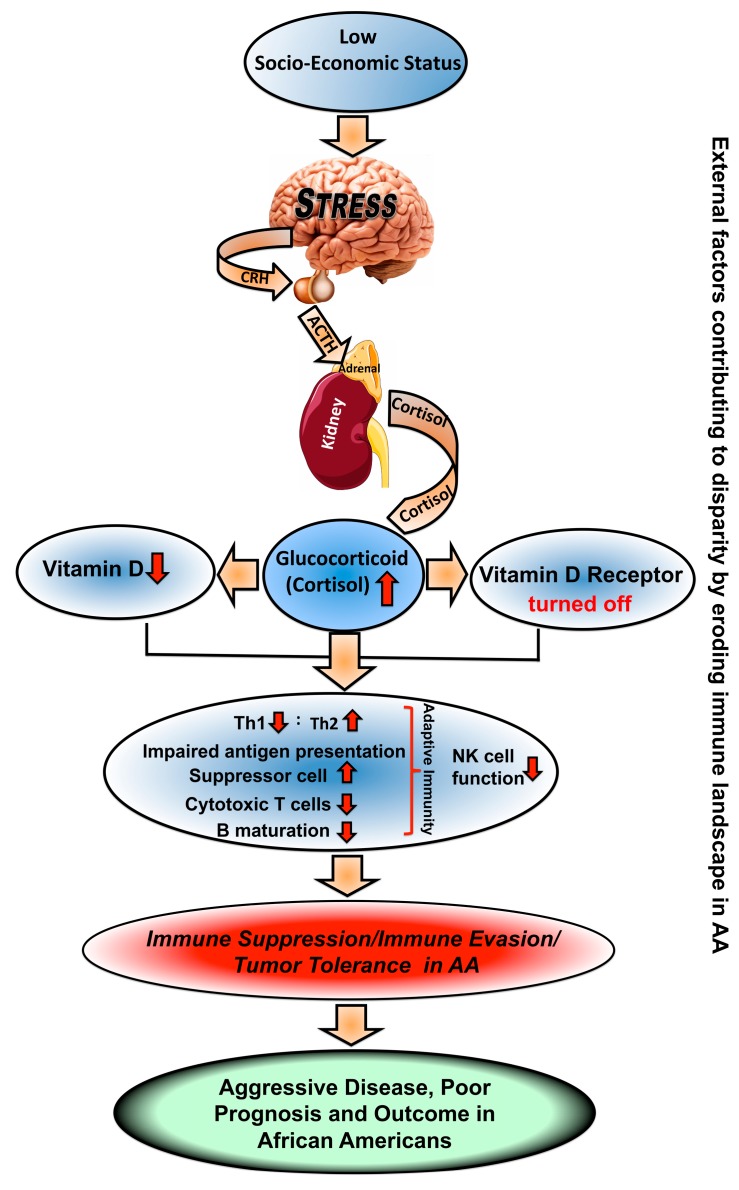
Immune landscape modifiers and cancer disparity: socioeconomic status-associated stress impacts on cortisol and Vitamin D and Vitamin D receptors. These are involved in eroding the immunological landscape by decreasing the Th1:Th2 ratio, impairing antigen presentation and NK cell function. Such changes favor aggressive disease and poor outcome in African American men. CRH, corticotropin releasing hormone; ACTH, adrenocorticotropic hormone.

Incidence and mortality associated with PCa is declining in the US, yet AAs continue to have higher mortality rates associated with aggressive disease. The growing literature reviewed in this article provides strong evidence that there is differential immune response among patients of different racial groups. Genetic predisposition in immune modifiers in AAs contributes to their poorer prognosis. Socio-economic status-associated differences in hormone profiles and cytokines involved in immune evasion and tumor tolerance in men of different descent might be significant contributors to racial disparity in PCa (Figure 2). Therefore, it is also imperative to consider the SES of participants of the clinical trial before concluding that immune-based therapies, such as sipuleucel-T, show better outcomes in AA PCa patients.
